# Supplementary material for: A Ratiometric Fluorescent Nano-Probe for Rapid and Specific Detection of Tetracycline Residues Based on a Dye-Doped Functionalized Nanoscaled Metal–Organic Framework
Source: Nanomaterials (Basel). 2019 Jul 4;9(7):976. doi: 10.3390/nano9070976 (PMC6669437; doi:10.3390/nano9070976)
Supplement: Supplementary file 1 [file nanomaterials-09-00976-s001.pdf]

Supplementary Materials

A Ratiometric Fluorescent Nano-Probe for Rapid and Specific Detection of Tetracycline Residues Based on a Dye-Doped Functionalized Nanoscaled Metal–Organic Framework

Lei Jia [1], Shengli Guo [1], Jun Xu [1,*], Xiangzhen Chen [1], Tinghui Zhu [1] and Tongqian Zhao [2,*]

[1] College of Chemistry and Chemical Engineering, Henan Polytechnic University, Jiaozuo 454000, China; jlxj@hpu.edu.cn (L.J.); guoshenglihpu@163.com (S.G.); cxzhpu@163.com (X.C.); zthhpu@163.com (T.Z.)

[2] Institute of Resources & Environment, Henan Polytechnic University, Jiaozuo 454000, China

* Correspondence: xjjl@hpu.edu.cn (J.X.); zhaotq@hpu.edu.cn (T.Z.); Tel.: +86-391-398-6816 (J.X.)

Table 1. The energy and the related counts for each element form EDX element analysis.

element	Energy (eV)	weight percentage
C	1257.337	35.29
O	816.009	11.53
Si	176.476	1.26
Cu	2359.465	28.15
Zr	883.720	23.59
Eu	38.015	0.16

Table S2 The color coordinates of CIE chromaticity diagram of Dye@UiO-66@SiO_2-Cit-Eu nano-probe for various concentrations of TC (from 0 to 6.0 μM).

TC concentrations(10^{-6}M)	0	0.1	0.2	0.4	0.6	0.9
x	0.168	0.177	0.191	0.205	0.221	0.243
y	0.121	0.121	0.132	0.135	0.143	0.147
TC concentrations(10^{-6}M)	1.2	1.6	2.0	3.0	4.0	6.0
x	0.271	0.293	0.323	0.364	0.401	0.428
y	0.166	0.169	0.187	0.208	0.219	0.237
